# Supplementary material for: Elucidation of Mechanical, Physical, Chemical and Thermal Properties of Microbial Composite Films by Integrating Sodium Alginate with Bacillus subtilis sp
Source: Polymers (Basel). 2021 Jun 26;13(13):2103. doi: 10.3390/polym13132103 (PMC8271853; doi:10.3390/polym13132103)
Supplement: Supplementary file 1 [file polymers-13-02103-s001.zip › polymers-1244976-supplementary.pdf]

polymers

MDPI

Supplementary Material

Elucidation of Mechanical, Physical, Chemical and Thermal Properties of Microbial Composite Films by Integrating Sodium Alginate with *Bacillus subtilis* sp.

Charles Ng Wai Chun [1], Husnul Azan Tajarudin [1,*], Norli Ismail [2,*], Baharin Azahari [3] and Muaz Mohd Zaini Makhtar [1]

[1] School of Industrial Technology, Bioprocess Technology Division, Universiti Sains Malaysia, 11800 Penang, Malaysia; charlesngwaichun@student.usm.my (C.N.W.C.); muazzaini@usm.my (M.M.Z.M.)

[2] School of Industrial Technology, Environmental Division, Universiti Sains Malaysia, 11800 Penang, Malaysia

[3] School of Industrial Technology, Bioresource, Paper and Coatings Division, Universiti Sains Malaysia, 11800 Penang, Malaysia; baharin@usm.my

* Correspondence: azan@usm.my (H.A.T.); norlii@usm.my (N.I.)

Citation: Wai Chun, C.N.; Tajarudin, H.A.; Ismail, N.; Azahari, B.; Mohd Zaini Makhtar, M. Elucidation of Mechanical, Physical, Chemical and Thermal Properties of Microbial Composite Films by Integrating Sodium Alginate with *Bacillus subtilis* sp. *Polymers* **2021**, *13*, 2103. https://doi.org/10.3390/polym13132103

Academic Editor: Hiroshi Ito

Received: 18 May 2021
Accepted: 28 May 2021
Published: 26 June 2021

Publisher's Note: MDPI stays neutral with regard to jurisdictional claims in published maps and institutional affiliations.

Copyright: © 2021 by the authors. Licensee MDPI, Basel, Switzerland. This article is an open access article distributed under the terms and conditions of the Creative Commons Attribution (CC BY) license (http://creativecommons.org/licenses/by/4.0/).

Table S1. Tensile strength, breaking strain and toughness of microbial composite films.

	Mass of Bacillus subtilis (g)	Triplicate	Tensile strength (MPa)	Breaking strain (%)	Toughness (MJ/m^3)
	**0**	**1**	0.4	85.644	0.004
		**2**	0.584	84.384	0.009
		**3**	0.849	83.088	0.034
**Average**			0.611	84.372	0.016
**Standard deviation**			0.226	6.906	0.016
	**0.1**	**1**	0.581	85.314	0.025
		**2**	0.626	84.35	0.015
		**3**	0.653	84.882	0.009
**Average**			0.62	84.849	0.016
**Standard deviation**			0.036	0.483	0.008
	**0.2**	**1**	0.753	84.65	0.02
		**2**	0.567	84.484	0.016
		**3**	0.585	86.144	0.019
**Average**			0.635	85.092	0.0183
**Standard deviation**			0.102	0.914	0.0021
	**0.3**	**1**	0.834	86.01	0.034
		**2**	0.68	85.048	0.022
		**3**	0.611	84.45	0.015
**Average**			0.709	85.169	0.024
**Standard deviation**			0.114	0.787	0.009
	**0.4**	**1**	0.776	82.39	0.022
		**2**	0.604	83.354	0.016
		**3**	0.829	89.832	0.035
**Average**			0.736	85.192	0.024
**Standard deviation**			0.118	4.047	0.009
	**0.5**	**1**	0.947	89.166	0.061
		**2**	0.756	85.812	0.03
		**3**	0.871	87.24	0.044
**Average**			0.858	87.406	0.0450
**Standard deviation**			0.096	1.683	0.016
	**0.6**	**1**	0.888	85.646	0.033
		**2**	0.776	85.878	0.022
		**3**	0.829	86.94	0.039
**Average**			0.831	86.155	0.0310
**Standard deviation**			0.056	0.69	0.009

Table S2. Thickness of microbial composite films.

Mass of Bacillis subtilis (g)	Triplicate	Readings of Precision Micrometer (mm)					Average (mm)	Overall Average (mm)
**0.0 g**	**1**	**0.505**	**0.604**	0.524	0.576	0.513	0.544	0.578
	2	0.583	0.636	0.581	0.617	0.642	0.612	
	3	0.661	0.516	0.528	0.587	0.601	0.578	
**0.1 g**	1	0.607	0.623	0.643	0.677	0.611	0.632	0.665
	2	0.618	0.703	0.692	0.687	0.717	0.683	
	3	0.762	0.603	0.702	0.656	0.677	0.680	
**0.2 g**	1	0.766	0.781	0.793	0.79	0.658	0.758	0.761
	2	0.689	0.776	0.782	0.743	0.762	0.750	
	3	0.801	0.812	0.793	0.762	0.699	0.773	
**0.3 g**	1	0.885	0.909	0.913	0.927	0.816	0.890	0.892
	2	0.805	0.912	0.973	0.873	0.991	0.911	
	3	0.943	0.947	0.868	0.796	0.818	0.874	
**0.4 g**	1	1.011	1.073	0.934	0.987	0.907	0.982	1.002
	2	0.963	0.998	1.087	1.106	0.977	1.026	
	3	0.936	0.948	1.044	1.087	0.976	0.998	
**0.5 g**	1	1.082	1.073	1.172	1.007	1.086	1.084	1.057
	2	1.073	1.088	1.074	1.096	0.992	1.065	
	3	0.976	1.067	1.082	1.012	1.006	1.029	
**0.6 g**	1	1.103	1.057	0.974	1.109	1.008	1.050	1.059
	2	1.063	0.969	1.043	1.101	1.048	1.045	
	3	1.17	1.112	1.051	1.008	1.072	1.826	

Table S3. Opacity (white light) of microbial composite films.

Mass of *Bacillus subtilis* (g)	Triplicate	Opacity (White light, %)	Average	High	Low	Standard deviation
**0**	0.1	7.46	7.52	7.73	7.39	0.18
	0.2	7.39				
	0.3	7.73				
**0.1**	1.1	7.83	8.1	8.28	7.83	0.24
	1.2	8.28				
	1.3	8.18				
**0.2**	2.1	8.3	8.39	8.44	8.3	0.07
	2.2	8.44				
	2.3	8.48				
**0.3**	3.1	10.31	9.19	10.31	8.49	0.98
	3.2	8.49				
	3.3	8.78				
**0.4**	6.1	15.7	12.16	15.7	10.02	3.09
	6.2	10.02				
	6.3	10.74				
**0.5**	5.1	13.43	13.65	14.15	13.38	0.43

	5.2	14.15				
	5.3	13.38				
**0.6**	4.1	16.76	16.45	16.76	16.21	0.28
	4.2	16.21				
	4.3	16.38				

Table S4. Opacity (black light) of microbial composite films.

Mass of *Bacillus subtilis* (g)	Triplicate	Opacity (Black light, %)	Average	High	Low	Standard deviation
**0**	0.1	38.88	38.41	39.14	38.09	0.64
	0.2	38.09				
	0.3	39.14				
**0.1**	1.1	37.9	37.86	38.44	37.34	0.6
	1.2	38.44				
	1.3	37.34				
**0.2**	2.1	37.92	38.61	38.98	37.92	0.6
	2.2	38.94				
	2.3	38.98				
**0.3**	3.1	43.03	39.29	43.03	36.92	3.28
	3.2	36.92				
	3.3	37.93				
**0.4**	6.1	50.56	41.69	50.56	36.13	7.77
	6.2	36.13				
	6.3	38.38				
**0.5**	5.1	39.99	40.55	50.56	39.99	0.52
	5.2	40.66				
	5.3	41.01				
**0.6**	4.1	41.12	41.18	42.07	40.36	0.86
	4.2	40.36				
	4.3	42.07				

Table S5. Brightness of microbial composite films.

		Readings						
**Mass of *Bacillus subtilis* (g)**	**Triplicate**	**1**	**2**	**3**	**Average**	**High**	**Low**	**Standard deviation**
0	0.1	4.23	4.14	4.06	4.24	4.76	3.9	0.25
	0.2	4.05	3.9	4.37				
	0.3	4.76	4.35	4.34				
0.1	1.1	4.23	4.14	4.06	4.24	4.76	3.9	0.25
	1.2	4.05	3.9	4.37				
	1.3	4.76	4.35	4.34				
0.2	2.1	7.06	6.52	6.62	6.65	7.06	6.24	0.27
	2.2	6.28	6.24	6.79				
	2.3	6.84	6,62	6.86				
0.3	3.1	6.14	7.2	6.54	6.67	7.3	5.57	0.54

	3.2	5.57	6.88	6.68				
	3.3	7.07	7.3	6.69				
0.4	4.1	7.38	8.99	5.22	7.38	8.99	5.22	1.17
	4.2	7.42	6.47	6.57				
	4.3	8.51	7.51	8.34				
0.5	6.1	7.32	8.23	8.48	7.88	8.48	7.32	0.49
	6.2	7.91	8.23	8.47				
	6.3	7.33	7.38	7.58				
0.6	5.1	10.62	9.26	9.2	8.19	10.62	6.64	1.32
	5.2	6.92	7.39	6.64				
	5.3	7.36	7.7	8.66				

Table S6. Conductivity of microbial composite films.

Mass of *Bacillus subtilis* (g)	Triplicate	Conductivity (mV)	Average (mV)
0	1	11	11.33
	2	10	
	3	13	
0.1	1	12	12
	2	13	
	3	11	
0.2	1	16	16.33
	2	15	
	3	18	
0.3	1	15	17.33
	2	19	
	3	18	
0.4	1	19	19.67
	2	19	
	3	21	
0.5	1	27	26
	2	26	
	3	25	
0.6	1	25	26.33
	2	28	
	3	26	

Table S7. Water absorption of microbial composite films.

Mass of *Bacillus subtilis* (g)	Triplicate	Weight of dried films (g)	Weight of wet films (g)	Percentage of water absorption (%)	Average (%)
0	1	0.07	0.26	271.43	264.29
	2	0.07	0.26	271.43	
	3	0.08	0.28	250	
0.1	1	0.07	0.27	285.71	269.05
	2	0.08	0.28	250	

	3	0.07	0.26	271.43	
0.2	1	0.08	0.29	262.5	270.83
	2	0.08	0.31	287.5	
	3	0.08	0.29	262.5	
0.3	1	0.08	0.3	275	279.17
	2	0.08	0.3	275	
	3	0.08	0.31	287.5	
0.4	1	0.08	0.31	287.5	289.35
	2	0.09	0.32	255.56	
	3	0.08	0.34	325	
0.5	1	0.08	0.34	325	300.93
	2	0.09	0.34	277.78	
	3	0.09	0.36	300	
0.6	1	0.09	0.37	311.11	307.78
	2	0.1	0.39	290	
	3	0.09	0.38	322.22	

Table S8. Thermogravimetric Analysis (TGA).

Step		Value	Sodium alginate film (Control)	Step	0.5 g microbial composite film
**1**	-0.79%, (-0.60 mg)	**Onset Temperature**	208.36 °C	-9.58% (0.52 mg)	211.21 °C
		**Residue**	70.11% (5.27 mg)		66.12% (3.62 mg)
		**Inflection Point**	211.99 °C		218.99 °C
		**Midpoint**	212.25 °C		221.95 °C
**2**	-27.25%, (-2.05 mg)	**Onset Temperature**	248.02 °C	-25.42% (-1.39 mg)	252.69 °C
		**Residue**	42.86% (3.22 mg)		40.69% (2.23 mg)
		**Inflection Point**	267.82 °C		270.44 °C
		**Midpoint**	267.73 °C		277.19 °C
**3**	-8.81%, (-0.66 mg)	**Onset Temperature**	417.09 °C	-6.21% (-0.34 mg)	431.08 °C
		**Residue**	34.05% (2.56 mg)		34.48% (1.89 mg)
		**Inflection Point**	436.80 °C		445.46 °C
		**Midpoint**	443.61 °C		466.16 °C
**4**	-8.15%, (-0.61 mg)	**Onset Temperature**	746.39 °C	-13.30% (0.73 mg)	657.30 °C
		**Residue**	25.75% (1.94 mg)		21.18% (1.16 mg)
		**Inflection Point**	777.03 °C		754.14 °C
		**Midpoint**	744.01 °C		701.79 °C
